# Automatic detection of punctate white matter lesions in infants using deep learning of composite images from two cases

**DOI:** 10.1038/s41598-023-31403-3

**Published:** 2023-03-17

**Authors:** Xuyang Sun, Tetsu Niwa, Takashi Okazaki, Sadanori Kameda, Shuhei Shibukawa, Tomohiko Horie, Toshiki Kazama, Atsushi Uchiyama, Jun Hashimoto

**Affiliations:** 1 Department of Radiology, Tokai University School of Medicine, 143 Shimokasuka, Isehara, 259-1193 Japan; 2 Department of Radiological Technology, Faculty of Health Science, Juntendo University, Bunkyo-Ku, Tokyo, Japan; 3 Department of Radiology, Tokai University Hospital, Isehara, Japan; 4 Department of Pediatrics, Tokai University School of Medicine, Isehara, Japan

**Keywords:** Computational biology and bioinformatics, Neurology

## Abstract

Punctate white matter lesions (PWMLs) in infants may be related to neurodevelopmental outcomes based on the location or number of lesions. This study aimed to assess the automatic detectability of PWMLs in infants on deep learning using composite images created from several cases. To create the initial composite images, magnetic resonance (MR) images of two infants with the most PWMLs were used; their PWMLs were extracted and pasted onto MR images of infants without abnormality, creating many composite PWML images. Deep learning models based on a convolutional neural network, You Only Look Once v3 (YOLOv3), were constructed using the training set of 600, 1200, 2400, and 3600 composite images. As a result, a threshold of detection probability of 20% and 30% for all deep learning model sets yielded a relatively high sensitivity for automatic PWML detection (0.908–0.957). Although relatively high false-positive detections occurred with the lower threshold of detection probability, primarily, in the partial volume of the cerebral cortex (≥ 85.8%), those can be easily distinguished from the white matter lesions. Relatively highly sensitive automatic detection of PWMLs was achieved by creating composite images from two cases using deep learning.

## Introduction

As magnetic resonance (MR) imaging is increasingly used in infants, milder white matter lesions, such as punctate white matter lesions (PWMLs), are often detected not only in preterm infants but also in term infants^1,2^. On MR imaging, PWMLs appear as small, focal, and often multiple lesions, predominantly in the periventricular white matter^3^. These lesions are visualized as small foci of high signal intensity on T1-weighted imaging and low signal intensity on T2-weighted imaging to the surrounding white matter^2,4^. Although PWMLs may be a result of mild injury^3,5^, heterogeneous etiologies of PWMLs have been considered, including punctate hemorrhage, subtle hypoxic-ischemic lesions, gliosis, genetic disorders, venous congestion, venous thrombosis, infection, and the presence of inborn errors of metabolism^3,4,6^. Although the relationship between PWMLs and neurodevelopmental outcomes remains controversial, an increased number of PWMLs, their presence in the corticospinal tract or frontal lobe, and large-volume PWMLs were reported to be related to adverse outcomes^3,7,8^. Therefore, precise and effective detection of PWMLs on MR imaging is important for infants in clinical practice.

Efficient image detection can be achieved by using currently developing techniques such as the deep learning method, which is based on a convolutional neural network (CNN)^9–14^. You Only Look Once v3 (YOLOv3; https://pjreddie.com/darknet/yolo/) is a CNN algorithm with an excellent network structure and fast object detection^15–18^. A typical deep learning method for image detection requires a considerable number of images for training. However, the collection of a sufficient number of training images may be difficult, particularly in pediatric radiology. By contrast, infants often undergo brain screening through MR imaging, with no abnormalities detected. Therefore, it is possible to collect many infant brain MR images without abnormality. In this context, we considered that the deep learning method for automatic detection of PWMLs may be accomplished by the use of composite images that combine MR images of infants with PWMLs and those without the abnormality. This study aimed to assess the automatic detectability of PWMLs in infants using deep learning of composite images.

## Materials and methods

### Participants

At our institution, preterm infants were examined through MR imaging at term-equivalent age for screening. Term infants (or late preterm–term infants) were also examined through MR imaging for some neurologic symptoms. From August 2013 to March 2021, MR images were reviewed for PWMLs. PWMLs were defined as small focal signal changes—increased signal intensity on T1-weighted imaging and decreased signal intensity on T2-weighted imaging to the surrounding white matter. First, a board-certified radiologist, with 21 years of experience in pediatric neuroradiology, reviewed MR images of infants and marked PWMLs. Another board-certified radiologist, with 12 years of experience in pediatric neuroradiology, confirmed the marked PWMLs. Differences in the presence of PWMLs were solved by consensus. Linear, nodular, or more widespread white matter lesions were excluded. Faint, dotted signals that could not be differentiated from noise on MR imaging were also excluded. Initially, 37 infants with PWMLs were selected, three of whom were subsequently excluded because thin-slice T1-weighted imaging was not obtained. Thus, the final analysis included 34 infants (gestational age, 26.3–39.1 weeks; mean ± standard deviation [SD], 34.1 ± 2.8 weeks). These 34 infants underwent MR imaging at the corrected gestational age of 37.1–40.9 weeks (38.8 ± 1.1 weeks). The number of PWMLs in these infants ranged from 1 to 19 (6.0 ± 5.4). No cases of severe motion artifact were present among these 34 infants with PWMLs.

During the same period, unexposed control infants who underwent MR imaging were also reviewed. Their MR images without abnormality were searched, and 158 infants were initially identified. Of these 158 cases, 74 infants were excluded because of motion artifact, and 12 infants were excluded because thin-slice T1-weighted imaging was not obtained. Thus, 72 infants (gestational age, 26.0–41.0 [32.9 ± 3.8]) without abnormal MR findings were eligible for this study. The examinations were conducted at the corrected gestational age of 36.7–46.3 [39.3 ± 1.6] weeks.

This study was approved by the Institutional Review Board for Clinical Research, Tokai University, and informed consent was waived because of the retrospective nature of the study (IRB No. 20R-363). This study was performed in accordance with the principles of the Declaration of Helsinki. The data used in this study were anomymized.

### MR imaging

The MR imaging was performed using 3T-MR units (Achieva Tx or Ingenia, Philips, Best, The Netherlands) with an eight-channel head coil for the Achieva Tx and a 12-channel head coil for the Ingenia during natural sleep. Sedating agents were not used for MR scanning. Infants were wrapped with a vacuum pillow to reduce their movements during MR scanning. A small silencer headphone was used. In this study, MR images for three-dimensional (3D) thin-slice T1-weighted gradient-echo images were analyzed, which were obtained with the following parameters: repetition time/echo time, 9.5/4.4 ms; inversion time, 1200 ms; matrix, 192 × 174; field of view, 160 × 143 mm; flip angle, 8°; slice thickness, 0.9 mm; average number, 1; slice number, 120; turbo factor (number of data samplings per shot), 200; parallel imaging sensitivity encoding factor, 1.5; and acquisition time, 2 min 52 s. The imaging resolution was 0.83 × 0.82 × 0.90 mm.

### Composite image

Composite images for deep learning were created by a combination of MR images of PWMLs and MR images without abnormality. First, the two infants with the highest number of PWMLs (19 and 17 PWMLs) were selected. Their PWML images were adjusted using Photoshop version 13.0 (Adobe, San Jose, CA, USA) using auto-contrast and auto-tone, and the PWMLs were selected using Photoshop auto-selection tools. The MR images without abnormalities were also adjusted using Photoshop auto-contrast and auto-tone before pasting the PWML images.

**Figure 1:**
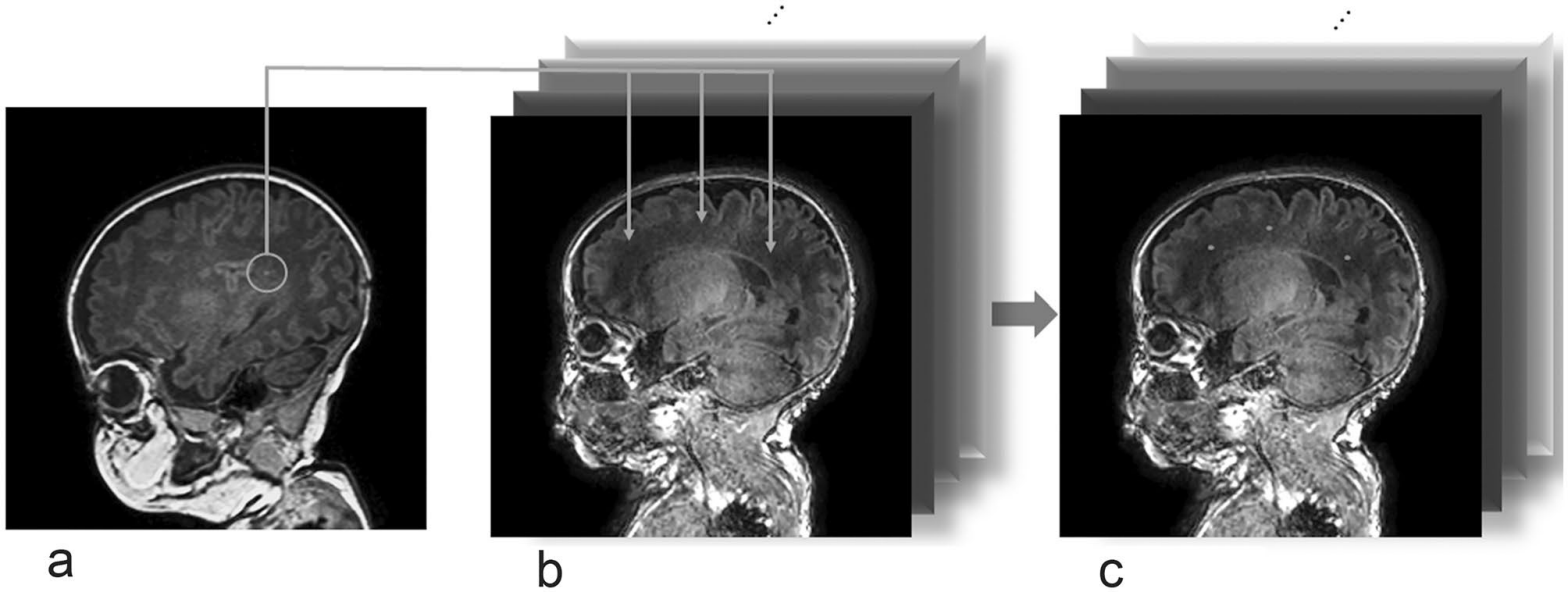
A scheme of a process to create composite images of punctate white matter lesions (PWMLs) for deep learning: PWMLs in an infant (**a**) are extracted, copied, and pasted onto T1-weighted images of infants without abnormal findings (**b**). Repeating this process creates many composite PWMLs images for the training of deep learning (**c**).

Next, the extracted PWML images were pasted onto the MR images without abnormality, which were randomly selected from the infants without abnormal MR findings at the following slices: the level of the most lateral portion, the posterior horn, and the trigone of the bilateral lateral ventricles. The PWMLs were pasted to MR images without abnormality in three regions from the frontal lobe to the parietal lobe in the white matter. The PWMLs in a specific MR imaging slice were copied to the randomly selected 72 MR imaging slices without abnormalities. The PWMLs from the two infants were shown in a total of 13 slices, resulting in these combinations for a total of 936 composite images. The PWMLs were manually pasted with Photoshop. Composite images were provided as 256 × 256 pixels (Fig. 1).

### Deep learning

To assess the differences among the number of training images, the composite images were randomly selected as a set of 100, 200, 400, and 600 images. Then, each set of composite images was augmented by applying left–right flip, scaling (0.95 and 1.05 magnification), and rotation (+ 15° and − 15°)^19,20^. Consequently, each set of 600, 1200, 2400, and 3600 composite images was provided.

The PWMLs on the composite images were labeled by rectangular bounding boxes using labelImg version 1.8.1 (https://github.com/tzutalin/labelImg). Each set of composite images was trained with an open-source library, ImageAI version 2.1.6 (https://github.com/OlafenwaMoses/ImageAI), based on Python version 3.7.6, Tensorflow version 2.4.0, and Keras version 2.4.3 using YOLOv3 on a personal computer (an Intel core i7-7820X central processing unit and an Nvidia GeForce RTX 3090 graphics processing unit). Composite images were divided into training and validation with a rate of 80%/20%^21^. The training was performed with a batch size of 16 and a maximum epoch of 1000.

### Image assessment

The PWMLs for 32 infants, which were not used for training, were assessed for automatic detection. Two board-certified radiologists, as previously described, conducted the evaluation as a reference for ground truth. Automatic detection for PWMLs was performed using the models after training. A threshold of detection probability of 20% was set for each training model. The detected locations and the values of detection probability were noted for each model. A resident radiologist who had 1 year of experience in general radiology but almost no experience in pediatric neuroradiology also assessed the PWMLs for comparison.

### Statistical analysis

Sensitivity and positive predictive value (PPV) were calculated for the automatic detection of PWMLs on each model trained with a set of 600, 1200, 2400, and 3600 composite images. Then, sensitivity and PPV for each training model were computed when the threshold of the detection probability was set to 30%, 40%, and 50%. The values for detection probability between true- and false-positive detections were compared using the Mann–Whitney test (the Shapiro–Wilk test did not reveal the normal distribution of the values for detection probability). False-positive detections were classified according to the location in the cerebral cortex/white matter. Sensitivity and PPV for the reading by the resident radiologist were also calculated.

Data were analyzed by using Excel version 2202 (Microsoft, Redmond, WA, USA) and MedCalc Statistical Software version 20.104 (MedCalc Software, Ostend, Belgium; https://www.medcalc.org). A *P* value of < 0.05 was considered statistically significant.

## Results

The time of training for each set of 600, 1200, 2400, and 3600 composite images was approximately 35.6, 72.2, 141.6, and 211.1 h, respectively. The number of PWMLs in the 32 infants for automatic detection ranged from 1 to 17 (5.1 ± 4.7). A total of 163 PWMLs were assessed for automatic detection by deep learning models. Mean average precision for the detection model trained by 600, 1200, 2400, and 3600 images were 0.5980, 0.8421, 0.8526, and 0.8546%, respectively.

**Figure 2:**
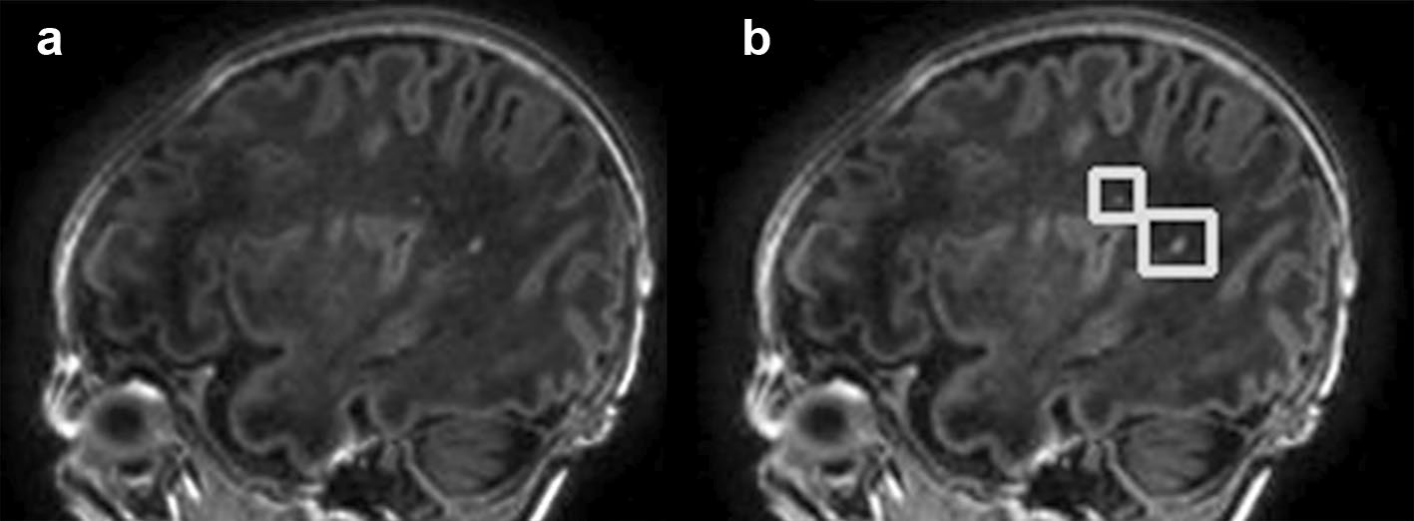
Automatic detection of punctate white matter lesions (PWMLs) in an infant: two PWMLs in a sagittal T1-weighted image (**a**) are correctly detected (**b**, squares).

Table 1 shows the sensitivity and PPV for detecting PWMLs according to each threshold of detection probability. Sensitivities decreased when the threshold of detection probability increased. Relatively high sensitivities (> 90%) were achieved with a threshold of detection probability of 20% and 30% for each training model (Fig. 2). Sensitivities for the detection model trained by 600 composite images were generally lower than those trained by 1200, 2400, and 3600 composite images. Few differences in sensitivities were noted among the models trained by 1200, 2400, and 3600 composite images.

**Table 1:** Sensitivity and positive predictive value for automatic detection of punctate white matter lesions by the threshold of detection probability for each training set of composite images.

Training sets for threshold of detection probability	Sensitivity	Positive predictive value
600 images		
20%	0.926	0.367
30%	0.908	0.559
40%	0.817	0.720
50%	0.390	0.928
1200 images		
20%	0.951	0.552
30%	0.945	0.550
40%	0.851	0.711
50%	0.623	0.815
2400 images		
20%	0.957	0.484
30%	0.945	0.684
40%	0.865	0.844
50%	0.589	0.980
3600 images		
20%	0.939	0.519
30%	0.926	0.659
40%	0.853	0.739
50%	0.638	0.904

The PPV varied greatly (0.367–0.980) according to the threshold of detection probability. The PPV increased according to the increment of the threshold of detection probability. Relatively high PPV (0.815–0.980) was shown at the threshold of detection probability of 50% for each training model, but low sensitivities were revealed at this threshold.

Median values (interquartile range) of the detection probabilities for PWMLs were 48.9 (44.8–53.0), 52.2 (47.0–56.9), 50.1 (44.2–54.2), and 52.0 (48.0–57.5) for training set of 600, 1200, 2400, and 3600 composite images, respectively; those for false-positive lesions were 28.7 (23.6–36.6), 37.8 (32.8–47.7), 27.6 (23.1–37.5), and 32.1 (24.6–44.6) for training set of 600, 1200, 2400, and 3600 composite images, respectively. The values of detection probability for PWMLs were significantly higher than those for false-positive lesions with each training set of composite images (*P* < 0.0001).

**Figure 3:**
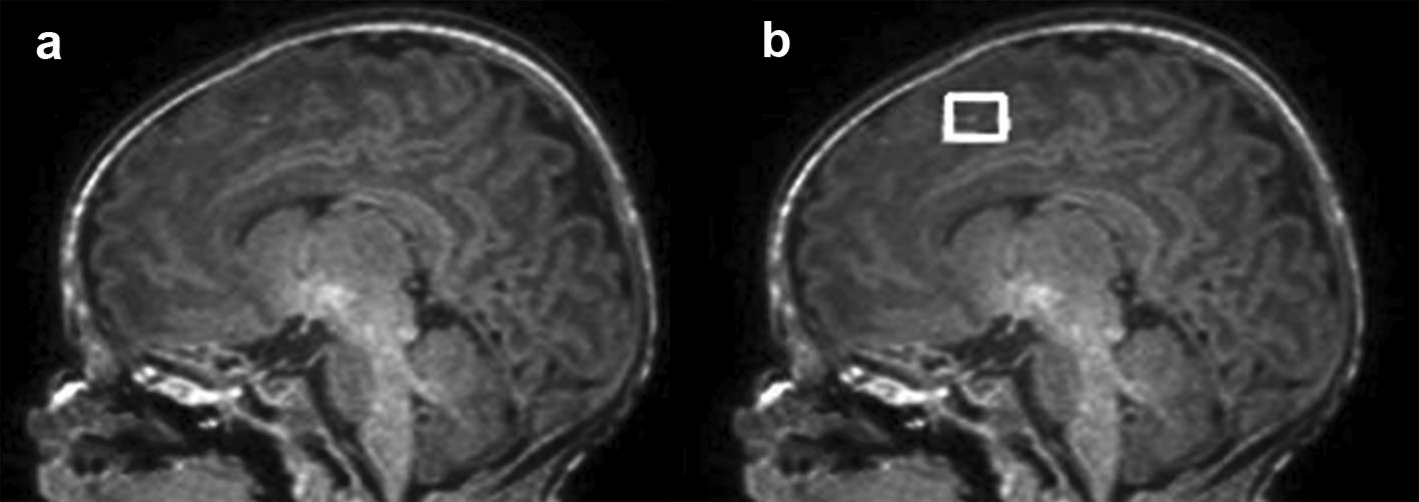
False-positive detection of punctate white matter lesions (PWMLs) in an infant. Automatic detection in (**b**) (square) appears to be a PWML but actually was a part of the cerebral cortex (**a**).

Table 2 shows the results for false-positive detection. The false-positive detections were primarily located in the cerebral cortex; the rate of false-positive detection in the cerebral cortex was more than 85.8% for all automatic detection models (Fig. 3). A relatively small number of false-positive detections (i.e., 0–8) were located in the cerebral white matter with the threshold of the detection probability greater than 30% for the training set of 1200, 2400, and 3600 composite images. The sensitivity and PPV for the resident radiologist reading of PWML detection were 0.730 and 0.967, respectively (Supplementary file).

**Table 2:** Location of false-positive detections by the threshold of detection probability for each training set of composite images.

Training sets for threshold of detection probability	Cerebral cortex detections (%)	Cerebral white matter detections (%)
600 images		
20%	224 (85.8)	37 (14.2)
30%	105 (89.7)	12 (10.3)
40%	50 (96.2)	2 (3.8)
50%	5 (100)	0 (0)
1200 images		
20%	118 (93.7)	8 (6.3)
30%	118 (93.7)	8 (6.3)
40%	56 (100)	0 (0)
50%	23 (100)	0 (0)
2400 images		
20%	115 (95.0)	6 (5.0)
30%	69 (97.2)	2 (2.8)
40%	26 (100)	0 (0)
50%	2 (100)	0 (0)
3600 images		
20%	124 (87.3)	18 (12.7)
30%	73 (93.6)	5 (6.4)
40%	47 (95.9)	2 (4.1)
50%	11 (100)	0 (0)

## Discussion

Study results showed relatively good automatic detection of PWMLs with the deep learning method using composite images. Generally, creating models for image detection on deep learning requires images from many patients, whereas our model achieved a relatively highly sensitive detection of PWMLs with composite images from only two infants. This method may be useful when images from many patients cannot be obtained to create an automatic image detection model by deep learning. In a previous report, Yi et al.^22^ showed the usefulness of automatic detection with the deep learning method by synthesized catheters on radiography, which showed that the method of synthesizing images may be effective to create an automatic detection model on deep learning.

The setting of the threshold of detection probability greatly affected the true- and false-positive detection rates. The lower threshold of detection probability resulted in highly sensitive PWML detection and high false-positive detection. By contrast, the higher threshold of detection probability resulted in a higher PPV and, simultaneously, a lower detectability of PWMLs. The values for detection probability were significantly higher for PWMLs than for false-positive lesions. Thus, the value for detection probability may be a factor to differentiate PWMLs from the false-positive lesions. It is important to note, however, that these values overlapped. Therefore, effective setting of the threshold for detection probability to discriminate between PWMLs and false-positive lesions appears to be difficult. Regarding the use of this model, we assume that sensitive detection would be important not to miss the detection of literally “punctate” white matter lesions in infants. From this viewpoint, the setting of the lower threshold of detection probability should be recommended. Although this approach induces more false-positive detections, most of the false-positive detections in our model were in the cerebral cortex. False-positive detection of a part of the cerebral cortex may not cause a serious obstacle to image interpretation because the separation of the cerebral cortex and white matter is simple in the usual process of image interpretation by observing the neighboring image slices. Therefore, the use of a lower threshold of detection probability, such as 30%, should be favored when applying this model in clinical practice. Meanwhile, false-positive detections occurred in the white matter, particularly at the setting of a lower threshold of detection probability. The cause of false-positive detections in the cerebral white matter was not fully elucidated; however, one cause may be noise on MR images. In such a case, false-positive detection may be reduced by improving MR imaging quality.

In this study, the detectability of PWMLs using a deep learning model was better for the training set of 1200, 2400, and 3600 composite images than that of 600 composite images, whereas PWML detectability did not seem to differ among the sets of 1200, 2400, and 3600 composite images. Therefore, the use of more composite images than 1200 images may achieve little to improve the detection model of deep learning. Generally, automatic image detection based on deep learning has a higher detectability with an increasing number of training images^23^. However, training with an increased number of composite images using the current method may be insufficient to improve the detectability of PWMLs, as a result of overtraining^9^. Considering the relatively long time of training, creating the detection model with many composite images may not be effective for the current method.

Effective detection of PWMLs is important for MR image interpretation in infants because the location and the number of PWMLs in this patient population were reported to be related to neurodevelopmental outcomes^5,8^. Recently, PWMLs have been well recognized because thinner slice images have been used in clinical practice as a result of the development of the MR scanner and the more frequent use of 3T-MR scanners in infants^3,24,25^. By contrast, the interpretation of images by radiologists has become more difficult because more images should be assessed. Accordingly, automatic detection by deep learning is expected to provide a synergetic effect with physicians, such as that achieved by using a second reader^12^. This system may aid in the precise and quick interpretation of MR images in clinical practice, particularly in the detection of a tiny lesion such as a PWML. In this study, our deep learning models sensitively detected PWMLs. This detection was better than that achieved by the resident radiologist. Therefore, this system can be used to assist PWML detection, particularly for physicians who have not yet gained sufficient experience with MR imaging for infants. In fact, several reports have described the improvement of lesion detectability through the collaboration between physicians and a deep learning system^12^. By contrast, although the current deep learning model did not reach high PPV, the resident radiologist’s reading revealed a high PPV. Therefore, combining this deep learning system with the resident radiologist’s reading would compensate for the resident’s learning curve. Further assessment of the interaction between physicians and deep learning systems should be evaluated in the future.

Although a relatively good detection model for PWMLs was achieved with our use of composite images, there is still room for improvement. Several issues regarding the model creation on deep learning must be considered to improve automatic PWML detection. First, we used the MR images of only two infants with PWMLs for training on deep learning because many MR images of PWMLs were not available. Additional use of PWML images to create more composite images may improve lesion detectability. Second, since creation of the composite images was originally devised, this method has not been fully examined. Other methods may be considered, such as pasting PWMLs with rotation or intensity modulations. Third, we performed data augmentation by horizontal flip, rotation, and magnification. However, other augmentation methods may be used, such as vertical flipping, rotation with a bigger angle, and noise addition^26^. Fourth, we set a relatively small square labeling to surround the PWMLs to create a deep learning model; however, the appropriate annotation for the method of labeling has not yet been well established^9^. Fifth, we used YOLOv3 to create training models on deep learning. Other networks, such as 3D U-net, GoogLeNet, a faster region-based convolutional neural network, and AlexNet, have been introduced for image detection^17,19,23,27,28^. We did not assess the differences in PWML detection among these networks in the present study. The relationship between these issues and their effects is complex and not yet understood. Therefore, further optimization of the deep learning model should be assessed in the future.

In this study, we achieved a relatively good automatic detection of PWMLs using the deep learning method. Another method for automatic lesion detection includes machine learning with a specific algorithm. We did not compare the detectability for PWMLs between the deep learning and machine learning methods; therefore, differences of detectability between these remain unknown. One possible advantage using our deep learning method is that it can be applied to other lesion detection, whereas machine learning requires a specific algorithm for detecting each lesion.

This study had several limitations. First, we assessed the MR images in a single MR acquisition method in a single institution. Reproducibility and generalizability should be assessed with other MR scanning methods and with other MR scanners. Second, the number of infants available for PWML detection was small. Additionally, the number of PWMLs in each infant varied. Although no cases with severe motion artifact occurred in infants with PWMLs, the cases included were not entirely free from motion artifact. We selected three slice levels of MR images of infants without abnormality to create composite images; however, the other combination pattern may be conceivable. These factors may have affected the study results. Third, we did not include faint hypersignals in the white matter suggestive of noise, although they could not completely be differentiated from tiny PWMLs. Fourth, the signal-to-noise ratio between the two MR scanners used in this study may have differed. Although the signal gain was corrected in each MR scanner, the differences in the signal-to-noise ratio may have affected the detectability of PWMLs and false-positive lesions. Fifth, we only assessed the detectability of PWMLs in this study. Further studies are required to evaluate whether this method can be applied for detection of other lesions.

## Conclusion

Providing composite images from two infants for a deep learning model achieved relatively highly sensitive automatic detection of PWMLs. The combination of this system in radiologic reading may contribute to the effective detection of PWMLs.

## Supplementary Information

Supplementary Information.

## Data Availability

The data used for analysis during this study are included in this published article and its supplementary information files. Image data generated and/or analyzed during the current study are not publicly available because of data privacy protection of patients but are available from the corresponding author on reasonable request.
